# Immunomodulatory potential of secretome from cartilage cells and mesenchymal stromal cells in an arthritic context: From predictive fiction toward reality

**DOI:** 10.3389/fmed.2022.992386

**Published:** 2022-10-12

**Authors:** Alessandra Colombini, Francesca Libonati, Silvia Lopa, Enrico Ragni, Paola De Luca, Luigi Zagra, Federico Sinigaglia, Matteo Moretti, Laura de Girolamo

**Affiliations:** ^1^Laboratorio di Biotecnologie Applicate all’Ortopedia, IRCCS Istituto Ortopedico Galeazzi, Milan, Italy; ^2^Cell and Tissue Engineering Laboratory, IRCCS Istituto Ortopedico Galeazzi, Milan, Italy; ^3^Hip Department, IRCCS Istituto Ortopedico Galeazzi, Milan, Italy; ^4^Regenerative Medicine Technologies Lab, Laboratories for Translational Research (LRT), Ente Ospedaliero Cantonale, Bellinzona, Switzerland; ^5^Department of Surgery, Service of Orthopaedics and Traumatology, Ente Ospedaliero Cantonale, Lugano, Switzerland; ^6^Faculty of Biomedical Sciences, Euler Institute, USI, Lugano, Switzerland

**Keywords:** adipose stem cells, bone marrow stem cells, secretome, miRNAs, early osteoarthritis, immunomodulation, cartilage cells

## Abstract

The purpose of the present study is to predict by bioinformatics the activity of the extracellular vesicle (EV)-embedded micro RNA (miRNAs) secreted by cartilage cells (CCs), adipose tissue-derived- (ASCs), and bone marrow-derived stem cells (BMSCs) and verify their immunomodulatory potential supporting our bioinformatics findings to optimize the autologous cell-based therapeutic strategies for osteoarthritis (OA) management. Cells were isolated from surgical waste tissues of three patients who underwent total hip replacement, expanded and the EVs were collected. The expression of EV-embedded miRNA was evaluated with the QuantStudio 12 K Flex OpenArray^®^ platform. Mientournet and ingenuity pathway analysis (IPA) were used for validated target prediction analysis and to identify miRNAs involved in OA and inflammation. Cells shared the expression of 325 miRNAs embedded in EVs and differed for the expression of a small number of them. Mienturnet revealed no results for miRNAs selectively expressed by ASCs, whereas miRNA expressed by CCs and BMSCs were putatively involved in the modulation of cell cycle, senescence, apoptosis, Wingless and Int-1 (Wnt), transforming growth factor beta (TGFβ), vascular endothelial growth factor (VEGF), Notch, Hippo, tumor necrosis factor alpha (TNFα), interleukin 1 beta (IL-1β), insulin like growth factor 1 (IGF-1), RUNX family transcription factor 2 (RUNX2), and endochondral ossification pathways. Cartilage homeostasis, macrophages and T cells activity and inflammatory mediators were identified by IPA as targets of the miRNAs found in all the cell populations. Co-culture tests on macrophages and T cells confirmed the immuno-modulatory ability of CCs, ASCs, and BMSCs. The study findings support the rationale behind the use of cell-based therapy for the treatment of OA.

## Introduction

Cell therapy for the treatment of early osteoarthritis (OA) is essentially based on mesenchymal stromal cells (MSCs), mainly adipose tissue-derived (ASCs), and bone marrow-derived stem cells (BMSCs). These cell sources represent the elective choice because adipose tissue and bone marrow are easily harvestable and allow an adequate cell number (1) to be obtained.

The rationale for the use of these cells lies in the fact that they are able to respond to the environment in which they are placed, acting as protagonists of immunomodulation if they have to face antagonists or a hostile microenvironment (2).

Autologous chondrocytes also represent a therapeutic option for the treatment of joint conditions with specific reference to focal chondral lesions (3). However, apart from their regenerative potential, chondrocytes show immunomodulatory abilities (4), likely explaining their clinical effectiveness also in patients with early OA (5–9).

Donor matched cartilage cells (CCs), ASCs and BMSCs were previously compared, in a published paper of our research group, in term of their phenotype and secretory features (10). All the analyzed cell types shared a similar immunophenotype, negative for hematopoietic markers and positive for mesenchymal stromal cell markers, and were able to differentiate into the osteogenic and chondrogenic lineages. Moreover, an exhaustive multiplex-based analysis of the cell secretome revealed that CCs exhibited the largest amount of secreted growth factors overall, with a special presence of chondrogenic, angiogenic, and pro-mitogenic molecules (10).

Characterizing the ideal candidates for cartilage cell therapy in osteoarthritic patients is fundamental to face in the best way the degenerative processes leading to tissue loss, as well as to counteract the inflammatory infiltration that represents a severe issue in an arthritic joint (11). In this regard, immune cells, particularly macrophages (65% of the infiltrate) (12–14) followed by T cells (22% of the infiltrate) (14–17), are recruited from the bloodstream and infiltrate into the synovium, participating to the chronicization of the inflammatory and catabolic processes leading to early OA (18, 19). In particular, macrophages have a multifaceted role in OA and their phenotypic alterations were observed during the pathology development, with M1 (pro-inflammatory) macrophages elevated in synovium and M2 (anti-inflammatory/remodeling) macrophages decreased (20). With respect to T cells, most of those found in the synovial membrane of patients with OA are helper T cells (CD4^+^), whereas cytotoxic T cells (CD8^+^) occur sparsely (21–23).

Using cell secretome and in particular extracellular vesicles (EVs) has been also indicated as a potential strategy to counteract OA (24). These vesicles carry proteins, nucleic acids and lipids playing important roles in the intercellular communication and representing useful biomarkers for physio-pathological conditions (25). EV-embedded micro RNAs (miRNAs) are small non-coding RNAs playing important roles in post-transcriptional regulation of biological processes even in cartilage (26). The expression profile of miRNA molecules is exploitable as a tool to have picture of normal and pathological tissues, searching for biomarkers of disease and therapy also in the OA context (27).

Considering the clinical relevance of cell-based therapies in OA treatment, with the aim of identifying the best cell candidate, the objective of the present study is to predict by bioinformatics the activity of the EV-embedded miRNAs secreted by CCs, ASCs, and BMSCs, and involved in OA and verify the immunomodulatory potential of these cells to support our bioinformatics findings. The knowledge of the interaction of these cells and immune cells will help to optimize the autologous cell-based therapeutic strategies for OA management.

## Materials and methods

### Isolation and expansion of cartilage cells, adipose tissue-derived and bone marrow-derived stem cells

This study was approved by the local Institutional Review Board (M-SPER-015). After patients’ informed written consent, articular cartilage harvested from superficial areas of femoral head/neck, bone marrow from femoral channel and subcutaneous adipose tissue from hip fat deposit were collected from 2 females (53 and 56 y/o) and 1 male (41 y/o) having OA (Kellgren–Lawrence III–IV), who underwent total hip replacement.

Cartilage cells and ASCs were isolated by enzymatic digestion, whereas BMSCs were selected for plastic adherence. All these cell types were characterized, as previously reported (10). Cells were seeded at a density of 5,000 cells/cm^2^, detached after 7 days and expanded for 14 days (2 passages). At passage 2 cells were frozen in liquid nitrogen using heat-inactivated FBS added with 10% (v/v) DMSO at concentration of 3–5 × 10^6^ cells/vial. After thawing, cells were cultured for other 7 days until passage 3.

Cartilage cells were cultured in high glucose DMEM supplemented with 10% FBS, 200 mM glutamine L-glutamine, 100 U/mL penicillin, 100 μg/mL streptomycin, 10 mM 4-(2-hydroxyethyl) piperazine-1-ethanesulfonic acid (HEPES), 1 mM sodium pyruvate (all reagents from Thermo Fisher Scientific Waltham, MA, USA). ASCs and BMSCs were cultured in α-MEM supplemented as described above, adding 5 ng/mL fibroblast growth factor 2 (FGF-2) (PeproTech, Rocky Hill, NJ, USA), to preserve their stemness features and proliferative potential (28, 29). Cells were maintained at 37°C, 5% CO_2_, and 95% humidity.

### Isolation of extracellular vesicles

Cartilage cells, ASCs, and BMSCs at passage 3 and at 90% confluence were washed with phosphate buffered saline (PBS) and serum free medium was added for 48 h. The culture supernatants (30 mL) were collected and differentially centrifuged at 4°C with the following steps: 376 × *g* for 15 min, 1,000 × *g* for 15 min, 2,000 × *g* for 15 min, 4,000 × *g* for 15 min, 4,000 × *g* for 15 min. The cleared supernatants were ultra-centrifuged at 100,000 × *g* for 3 h at 4°C in a 70 Ti rotor (Beckman Coulter, Pasadena, CA, USA) to obtain EVs. EV pellets were suspended in 100 μL PBS, counted and characterized in term of size, shape, and surface marker expression, as previously reported (30–32). Cell viability after 48 h in serum free medium was checked with a NucleoCounter NC-3000 (ChemoMetec, Allerød, Denmark) to verify that culture in serum free medium did not compromise cell viability. CCs showed a viability of 95.1 ± 0.5%, BMSCs of 96.5 ± 2.0%, and ASCs of 97.2 ± 1.4%.

### Expression of extracellular vesicles-embedded micro RNA

The EV pellets were dissolved by Trizol and low molecular weight nucleic acids (<200 nt) were obtained with miRNeasy Kit and RNeasy CleanUp Kit (Qiagen, Hilden, Germany). During the extraction, synthetic ath-miR-159a was added as a spike-in to each sample as quality control for the process.

Reverse transcription and pre-amplification were performed to obtain cDNAs to be used as template for Real-Time PCR with the QuantStudio 12 K Flex OpenArray^®^ Platform (QS12KFlex). The Open Array covered 754 human miRNA sequences from the Sanger miRBase v21 Gene, divided into A and B panels.

### micro RNA data normalization

The miRNA expression was analyzed by Expression Suite Software (Life Technologies, Carlsbad, CA, USA). The spike-in was used to equalize A and B panels of the Open Array and to balance any technical difference during the process (33). C_*RT*_ of 27 was considered as a threshold for the presence/absence of amplification. Global mean, calculated from miRNAs amplified in all samples, was the normalization method (34). The relative quantification 2^–Δ*CRT*^ was used to determine the miRNA expression.

### micro RNA target prediction analysis

The bioinformatics Mientournet tool (35)^1^ was used for validated target prediction analysis, considering miRTarBase database for experimentally validated miRNA-target interactions. The analysis specifically focused on miRNA embedded in the EVs from CCs, ASCs, or BMSCs.

Ingenuity Pathway Analysis (IPA; Ingenuity^®^ Systems, Redwood City, CA, USA)^2^ was used to identify, among all the analyzed miRNAs, the ones experimentally observed as involved in OA. To achieve this, the miRNA Target Filter tool was applied as follows: “experimentally observed” and disease “skeletal and muscular disorders,” especially involved in “osteoarthritis pathway,” or “inflammatory response.”

Relevant pathways for immune response and OA were identified by the IPA tool using the target genes of miRNA of the three cell populations.

### Cell modulation of macrophages switch

Human peripheral blood mononuclear cells were isolated by Ficoll (GE Healthcare, Chicago, IL, USA) density gradient separation from 16 buffy coats of healthy donors obtained from the local blood bank. Monocytes were then isolated using CD14 magnetic microbeads (MACS, Miltenyi, Bergisch Gladbach, Germany) (36). After isolation, monocytes were counted and frozen in liquid nitrogen using heat-inactivated FBS added with 10% (v/v) DMSO at concentration of 10 × 10^6^ cells/vial.

After thawing, 90 × 10^6^ pooled monocytes were seeded at a density of 2 × 10^5^ cells/cm^2^ in RPMI 1640 (Gibco, St. Louis, MO, USA) added with 10% heat-inactivated FBS, 100 U/mL penicillin, 100 μg/mL streptomycin, 200 mM glutamine (Thermo Fisher Scientific, Waltham, MA, USA). The strategy to pool monocytes was used to achieve a suitable cell number for the following tests. To differentiate monocytes into M0 macrophages, 20 ng/mL of macrophage colony-stimulating factor (M-CSF, Peprotech Inc., Rocky Hill, NJ, USA) was added to the medium (37–39). The medium was refreshed every 2/3 days until day 9. In parallel, at day 2, CCs, ASCs, and BMSCs from the three matched donors were thawed and plated on polycarbonate membrane of *trans*-wells (Merck, Darmstadt, Germany) at a density of 0.7 × 10^5^ cells/*trans*-well and left in appropriate expansion medium to favor cell adhesion. At day 7, *trans*-wells seeded with CCs, ASCs, or BMSC were transferred to the macrophage plates. During the co-culture phase, which lasted 2 days, macrophage culture medium and the appropriate expansion medium of CCs, ASCs, and BMSC were combined in a 1:1 ratio. Non-co-cultured M0 macrophages were used as control.

After 2 days of co-culture, the macrophage immunophenotype was analyzed by flow cytometry. Briefly, macrophages were washed with PBS, detached with non-enzymatic cell dissociation buffer (Thermo Fisher, Frankfurt, Germany) and centrifuged at 500 × *g* for 5 min to collect them.

Macrophages were then suspended in MACS buffer (Miltenyi Biotec, Bergisch Gladbach, Germany), treated with FcR Blocking Reagent (Miltenyi Biotec, Bergisch Gladbach, Germany) for 10 min at 4°C to block unwanted binding of antibodies to human Fc receptor and counted. Afterward, 10^5^ cells were stained to evaluate the expression of cell surface markers with the following antibodies: anti-human CD80-APC (Clone REA661, Miltenyi Biotec, Bergisch Gladbach, Germany) and CCR7-APC/Fire-750 (Clone G043H7, Biolegend, San Diego, CA, USA) for M1 phenotype, anti-human CD206-FITC (Clone 15–2, Biolegend, San Diego, CA, USA) for M2a phenotype, and anti-human CD163-PE (Clone GHI/61, Biolegend, San Diego, CA, USA) for M2c phenotype. Unstained cells were used as negative control. All the stains were performed at 4°C for 20 min in the dark. Data were acquired using a Cytoflex flow cytometer (Beckman Coulter, Brea, CA, USA) acquiring a minimum of 10,000 events.

### Characterization of T cells after co-culture

After isolation by Ficoll, 2 × 10^5^ human peripheral blood cells (PBMCs) were co-cultured with 1 × 10^5^ or 2 × 10^4^ CCs, ASCs, and BMSCs, plated 2 days before. After 4 days of co-culture, PBMCs were collected and stained with monoclonal anti-human CD3-APC antibody (Clone UCHT1, Biolegend, San Diego, CA, USA) for gaiting lymphocytes and with monoclonal anti-human CD4-PE/Cy7 antibody (Clone RPA-T4, Biolegend, San Diego, CA, USA), and monoclonal anti-human CD8-PerCP antibody (Clone SK1, Biolegend, San Diego, CA, USA) to evaluate the ability of cells to modify the CD4^+^/CD8^+^ T cells ratio. Cells were analyzed on a Cytoflex flow cytometer acquiring 10,000 events.

### Statistical analysis

To analyze the data obtained from tests on macrophages and T cells, the normality of data distribution was assessed by Kolmogorov–Smirnov test. Unpaired Student’s *t*-test was used to compare control cells and co-cultured cells. Significance difference was considered for *p* ≤ 0.05. Statistical analysis was performed using GraphPad software (GraphPad Prism v5.00, La Jolla, CA, USA).

## Results

### Cartilage cells, adipose tissue-derived and bone marrow-derived stem cells share the expression of most micro RNAs

Among all the 428 detected miRNAs, 325 were embedded in the EVs from all the 3 cell populations, 26 were embedded in the EVs from CCs and ASCs, 21 were embedded in the EVs from CCs and BMSCs, and 17 were embedded in the EVs from ASCs and BMSCs. The miRNAs selectively embedded in the EVs from only one cell population were 16 from CCs, 12 from ASCs, and 11 from BMSCs. The list of all the analyzed miRNAs and their C_*RT*_ in each single cell population is showed in Supplementary Table 1.

**FIGURE 1:**
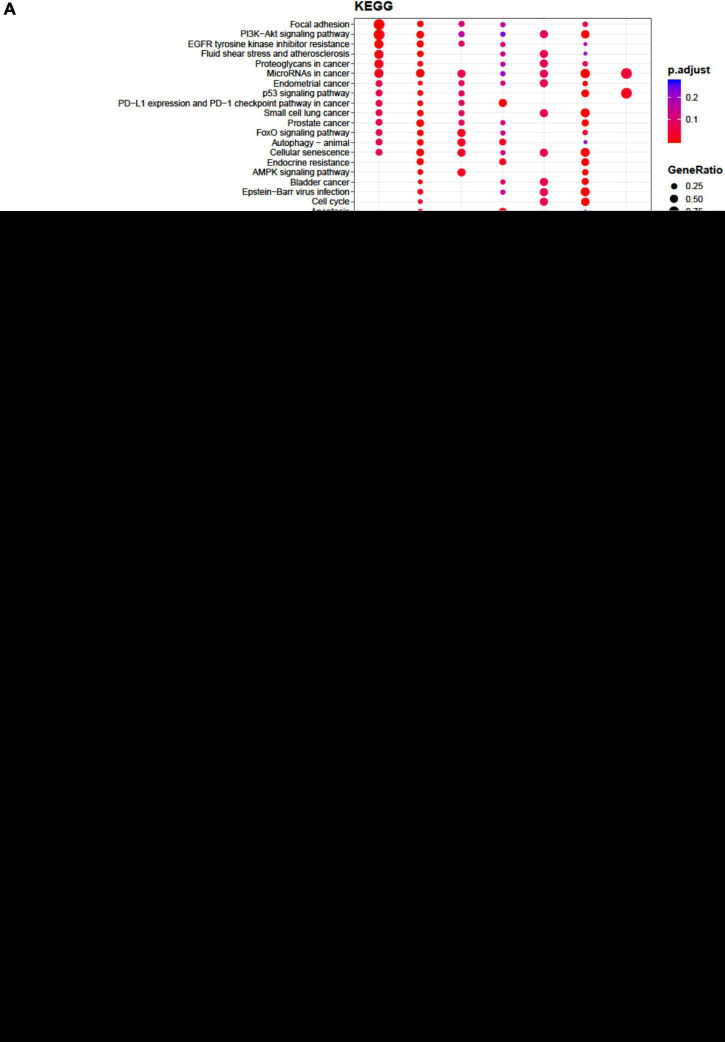
Functional enrichment analysis on micro RNAs (miRNAs) selectively expressed by cartilage cells (CCs). This analysis was conducted by KEGG pathway **(A)**, Reactome **(B)**, and Wikipathway **(C)**. Significant pathways are listed and represented by circles colored according to the significance of the enrichment and their size is proportional to the number of target genes regulating the described signaling pathways.

**FIGURE 2:**
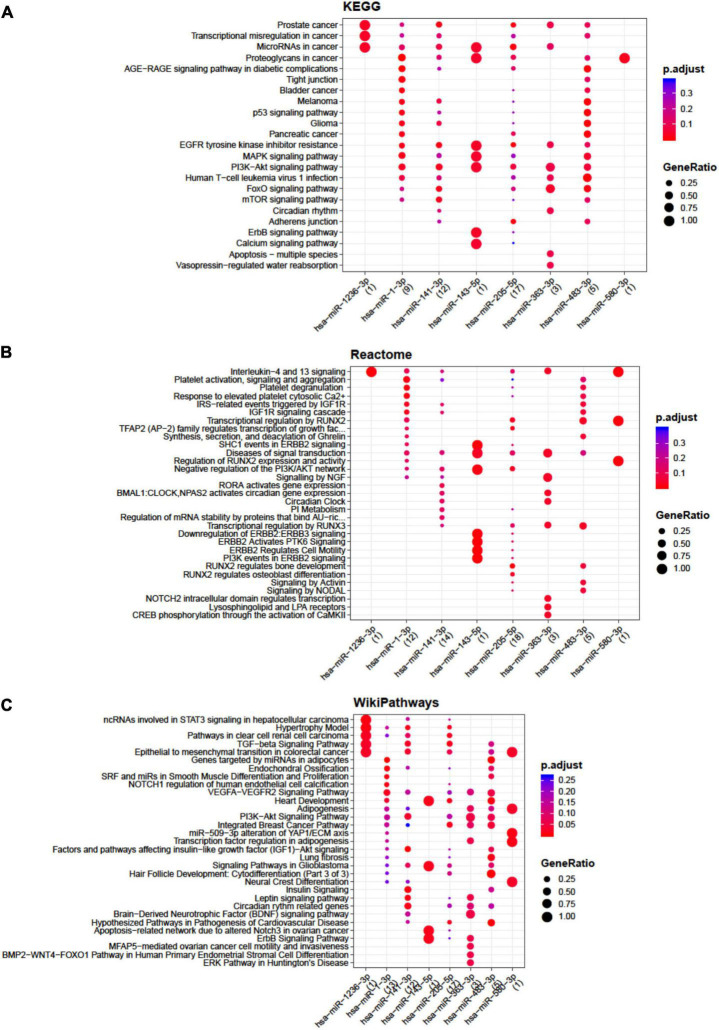
Functional enrichment analysis on micro RNAs (miRNAs) selectively expressed by bone marrow-derived stem cells (BMSCs). The analysis was conducted by KEGG pathway **(A)**, Reactome **(B)**, and Wikipathway **(C)**. Significant pathways are listed and represented by circles colored according to the significance of the enrichment and their size is proportional to the number of target genes regulating the described signaling pathways.

The lists of miRNAs embedded only in the EVs from each single cell population with target genes were retrieved by setting at least two gene-miRNA interactions (Supplementary Tables 2–4) as a threshold. Functional enrichment analysis was conducted by the open-source tools KEGG pathway, Reactome, and Wikipathway (Figures 1, 2). Significant pathways overrepresented within the targets of selected miRNAs are listed and indicated by circles, starting with those that are common to at least two miRNAs. Circles are colored according to the significance of the enrichment and their size is proportional to the number of involved targets. Bioinformatics analysis evidenced that no relevant pathways were modulated by miRNAs embedded only in the EVs from ASCs, whereas some relevant pathways were modulated by miRNAs embedded in the EVs from CCs and BMSCs. In particular, for CCs miR-17-3p, miR-25-5p, miR-200c-3p, and miR-449a showed the highest number of interactions (Supplementary Table 2). These miRNAs putatively modulate cell cycle, senescence, apoptosis, Wingless and Int-1 (Wnt), transforming growth factor beta (TGFβ), vascular endothelial growth factor (VEGF), Notch, Hippo, tumor necrosis factor alpha (TNFα) and interleukin 1 beta (IL-1β) signaling (Figure 1), potentially related to OA.

Concerning BMSCs, the highest number of interactions was showed by miR-141-3p, miR-143-5p, miR-363-3p, miR-205-5p, and miR-483-3p (Supplementary Table 4) mainly involved in apoptosis, TGFβ, insulin like growth factor 1 (IGF-1), RUNX family transcription factor 2 (RUNX2), and endochondral ossification pathways (Figure 2).

All the miRNAs embedded in the EVs from all the three populations of cells were analyzed by IPA tool to identify their involvement in OA and inflammation (Table 1). None of the miRNAs embedded only in the EVs from CCs was involved in OA. Six miRNAs (miR-140-5p, 302a-3p, 101-3p, 138-5p, 24-3p, and 126-3p) appeared up-regulated in CCs in comparison with both ASCs and BMSCs. Additional 13 miRNAs (miR-210, miR-532-5p, miR-196b-5p, miR-335-5p, miR-203, miR-21-5p, miR-100-5p, miR-615-5p, miR-185-5p, miR-186-5p, miR-130a-3p, let-7a-5p, and miR-133a) were up-regulated in the EVs from CCs in comparison with ASCs, whereas only 4 miRNAs (miR-1285-3p, miR-487b, miR-155-5p, and miR-9-5p) were up-regulated in the EVs from CCs in comparison with BMSCs.

**TABLE 1:** micro RNAs (miRNAs) in the extracellular vesicle (EVs) from cartilage cells (CCs), adipose tissue-derived (ASCs), and bone marrow-derived stem cells (BMSCs) and their target genes identified as involved in inflammation or osteoarthritis (OA) (target genes in blue) by ingenuity pathway analysis (IPA).

miRNA	Fc vs. ASCs	Fc vs. BMSCs	Target genes
miR-140-5p	**36.6**	**5.4**	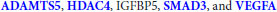
miR-302a-3p	**8.4**	**4.0**	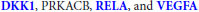
miR-101-3p	**4.0**	**3.7**	**PTGS2**
miR-138-5p	**3.5**	**3.2**	ROCK2
miR-24-3p	**2.1**	**2.0**	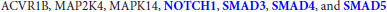
miR-126-3p	**2.0**	**2.5**	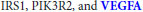
miR-210	**10.8**	1.7	ACVR1B
miR-532-5p	**9.8**	1.0	RUNX3
miR-196b-5p	**8.9**	0.7	**S100A9**
miR-335-5p	**7.8**	1.0	PTPN11, PXN, RASA1, and SRF
miR-203	**6.9**	1.7	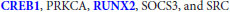
miR-21-5p	**4.5**	1.7	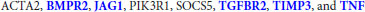
miR-100-5p	**4.3**	1.0	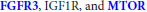
miR-615-5p	**3.1**	1.3	IGF1R
miR-185-5p	**2.6**	1.0	AKT1, CDC42
miR-186-5p	**2.3**	1.0	FOXO1
miR-130a-3p	**2.0**	1.0	**SMAD4**
miR-1285-3p	0.6	**8.2**	AKT2
miR-487b	1.4	**3.2**	MAP2K4
miR-155-5p	1.9	**2.3**	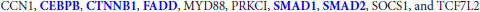
miR-191-5p	1.9	1.4	IL6
miR-31-5p	1.3	1.6	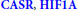
miR-214-3p	1.0	1.0	**ATF4**
miR-34a-5p	0.9	0.6	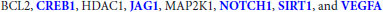
miR-29a-3p	1.6	1.3	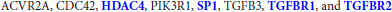
miR-26a-5p	1.5	1.4	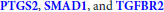
miR-27a-3p	1.8	1.4	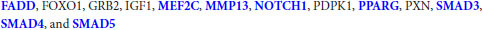
miR-132-3p	0.8	0.7	**MMP9**
miR-22-3p	1.6	1.1	BMP7, SRF
miR-25-3p	1.7	1.2	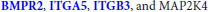
miR-23a-3p	1.8	1.2	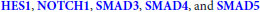
miR-19a-3p	1.1	0.8	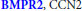
miR-125a-5p	0.6	0.6	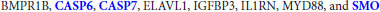
miR-128	1.7	1.0	**TGFBR1**
miR-491-5p	1.6	1.4	BCL2L1
miR-222-5p	0.8	0.7	ACTA2, ROCK2
miR-18a-5p	1.2	0.6	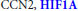
miR-199a-5p	0.9	0.7	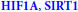
miR-15a-5p	1.7	0.9	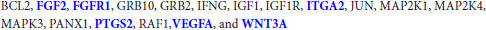
miR-1271-5p	1.4	1.8	FOXO1, IRS1
miR-335-3p	1.5	0.8	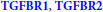
miR-139-5p	1.3	0.8	FOXO1, IGF1R, and SHC1
miR-150-5p	1.0	1.0	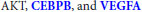
miR-184	1.2	1.2	AKT2
miR-223-3p	0.8	0.7	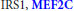
miR-296-3p	0.8	1.3	**CREB1**
miR-193a-3p	0.8	0.9	PTK2, RPS6KB2
miR-221-3p	**0.4**	**0.3**	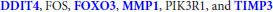
miR-149-5p	**0.3**	**0.5**	RAP1A, RAP1B
miR-218-5p	**0.1**	**0.1**	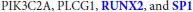
miR-143-3p	**0.5**	**0.1**	BCL2, IGFBP5, KRAS, and MAPK12
miR-181a-5p	**0.2**	**0.1**	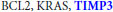
miR-197-3p	**0.4**	**0.5**	ACVR1
miR-296-5p	**0.5**	**0.3**	BCL2
miR-503	**0.1**	**0.4**	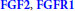
miR-542-3p	**0.4**	**0.5**	**PTGS2**
miR-145-5p	**0.4**	**0.1**	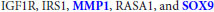
miR-124-3p	**0.02**	0.9	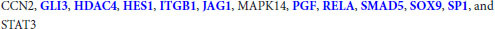
miR-204-5p	**0.2**	0.6	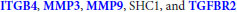
miR-146a-5p	**0.3**	0.6	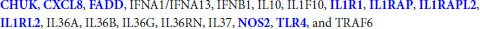
miR-422a	**0.4**	0.6	CASP9, IGF1R, and PDPK1
miR-34b-5p	**0.5**	0.7	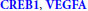
miR-142-3p	0.6	**0.3**	BCL2L1, PRKCA
miR-7-5p	1.1	**0.3**	FOS, IRS1, IRS2, p70 S6k, and RAF1
miR-106a-5p	0.7	**0.5**	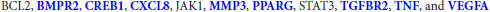
miR-193a-5p	0.6	**0.5**	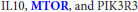
miR-324-5p	1.2	**0.5**	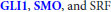
miR-30a-3p	1.1	**0.5**	CCN1, PIK3C2A
let-7a-5p	**2.6**	**0.5**	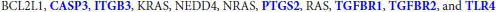
miR-9-5p	**0.2**	**2.3**	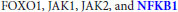
miR-133a	**2.3**	/	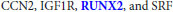
miR-486-5p	1.1	/	FOXO1
miR-122-5p	1.7	/	AKT3 MAPK11
miR-200b-3p	0.8	/	PLCG1
miR-135b-5p	/	0.9	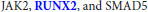
**miRNA**	**CRT ASCs**	**CRT BMSCs**	**Target genes**
miR-125b-1-3p	20.9	21.3	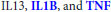
miR-129-5p	22.7	22.5	**BMPR2**
miR-141-3p	22.5		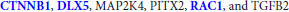
miR-18a-3p	25.9	26.2	KRAS
miR-205-5p	18.8		**VEGFA**
miR-219-5p		24.5	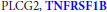
miR-375	23.8	24.9	JAK2, PDPK1
miR-483-3p	19.3		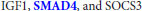

In bold fold change Fc ≥ 2 or ≤0.5 of expression in CCs in comparison with ASCs and BMSCs considered of interest.

Ten miRNAs (miR-221-3p, miR-149-5p, miR-218-5p, miR-143-3p, miR-181a-5p, miR-197-3p, miR-296-5p, miR-503, miR-542-3p, and miR-145-5p) were down-regulated in the EVs from CCs in comparison with both ASCs and BMSCs. Additional six miRNAs (miR-124-3p, miR-204-5p, miR-146a-5p, miR-422a, miR-34b-5p, and miR-9-5p) were down-regulated in the EVs from CCs in comparison with ASCs, whereas seven miRNAs (miR-142-3p, miR-7-5p, miR-106a-5p, miR-193a-5p, miR-324-5p, miR-30a-3p, and let-7a-5p) were down-regulated in the EVs from CCs in comparison with BMSCs.

Finally, four miRNAs (miR-125b-1-3p, miR-129-5p, miR-18a-3p, and miR-375) in the EVs from ASCs and BMSCs, three in the EVs from ASCs (miR-141-3p, miR-205-5p, and miR-483-3p), and one in the EVs from BMSCs (miR-219-5p) were involved in OA and not expressed in the EVs from CCs.

**FIGURE 3:**
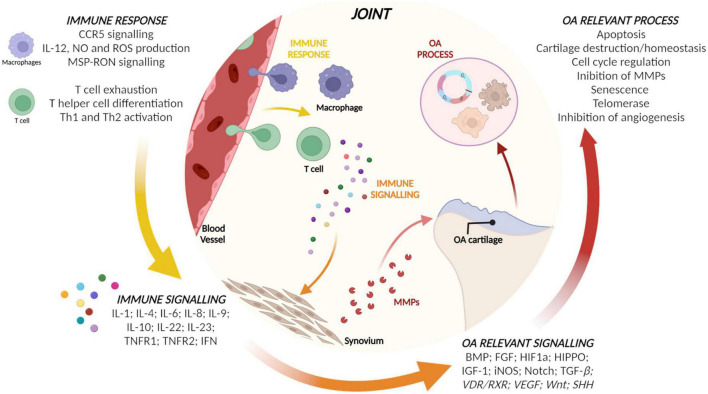
Relevant pathways for immune response and osteoarthritis (OA) identified by ingenuity pathway analysis (IPA) using the target genes of micro RNAs (miRNAs) of the three populations.

Micro RNAs in the EVs from CCs, ASCs, and BMSCs and their target genes identified as involved in inflammation or OA by IPA are reported in Table 1. Starting from target genes retrieved, relevant pathways for immune response and OA were identified by IPA and reported in Figure 3. Briefly, macrophages and T cells as actors and pro- and anti-inflammatory cytokines and mediators were identified as modulated by target genes of miRNAs of interest for what concerns immune response and signaling. Finally, cartilage homeostasis and cell proliferation-related pathways involved in OA modulated by the identified target genes were observed.

### Cartilage cells, adipose tissue-derived and bone marrow-derived stem cells potential in macrophage polarization

**FIGURE 4:**
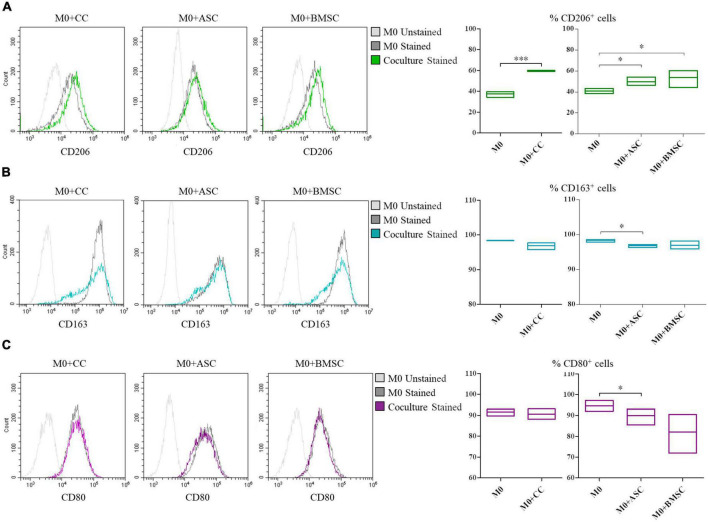
Modulation of M0 macrophages polarization by cartilage cells (CCs), adipose tissue-derived (ASCs), and bone marrow-derived stem cells (BMSCs). For CD206^+^ **(A)**, CD163^+^ **(B)**, and CD80^+^ **(C)** cells representative plots overlay show M0 co-cultured with the different cell types. Floating bars represent mean to max percentages and mean values of cells positive for the different analyzed markers in M0 and co-cultured M0 with CCs, ASCs, and BMSCs. *N* = 3; *p < 0.05, ^***^p < 0.0001.

Cartilage cells, ASCs, and BMSCs co-cultured with macrophages shared the ability to promote the increase of the CD206^+^ M2a anti-inflammatory macrophages (1.6, 1.2, and 1.4-fold increase, respectively; *p* < 0.0005 for CCs and *p* ≤ 0.05 for MSCs), Figure 4A. ASCs showed a decrease (*p* < 0.05) of CD163^+^ M2c remodeling macrophages (Figure 4B) and of CD80^+^ M1 inflammatory macrophages (Figure 4C).

### Cartilage cells, adipose tissue-derived and bone marrow-derived stem cells modulation of T cell survival and phenotypes

**FIGURE 5:**
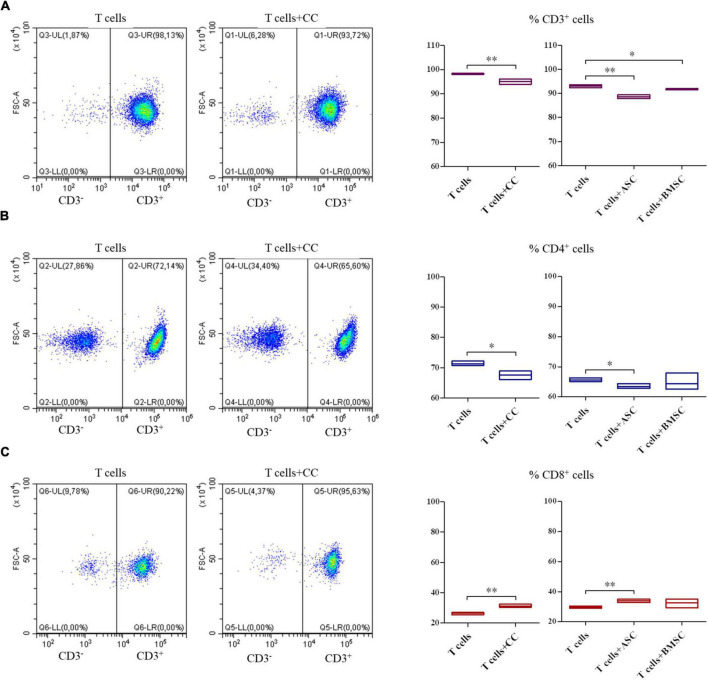
Modulation of T cell survival and phenotype by cartilage cells (CCs), adipose tissue-derived (ASCs), and bone marrow-derived stem cells (BMSCs). For CD3^+^ **(A)**, CD4^+^ **(B)**, and CD8^+^ **(C)** cell representative density plots show T cells co-cultured with CCs. Floating bars represent mean to max percentages and mean values of cells positive for the different analyzed markers in T cells and co-cultured T cells with CCs, ASCs, and BMSCs. *N* = 3; *p < 0.05, ^**^p < 0.001.

The co-culture of all the three cell types and PBMCs showed a decrease in the CD3^+^ T lymphocytes survival (Figure 5A, *p* < 0.01 for CCs and ASCs and *p* < 0.05 for BMSCs). When looking at T cell phenotype, a decrease in CD4^+^ T cells survival was observed in CCs and ASCs (*p* < 0.05) (Figure 5B). On the contrary, CD8^+^ T cells percentage increased in presence of CCs and ASCs (*p* < 0.01) (Figure 5C).

## Discussion

The main findings of the present investigation show that there was a similar basal expression of EV-embedded miRNAs in the CCs and both types of MSCs. In general, cartilage homeostasis-related pathways are identified as targets of the miRNAs found in all the cell populations. Interestingly, macrophages and T cells are actors potentially modulated by miRNAs in the EVs from all the three cell types. Donor-matched CCs and MSCs behave similarly in the modulation of the anti-inflammatory macrophages polarization and of the T cell survival and phenotypes. Each cell population presents specific properties to be exploited to treat early OA. Of the 3 cell populations, ASCs seem to be the most anti-inflammatory, but the least pro-remodeling ones, whereas CCs and BMSCs show a more prominent ability of inflammatory modulation and remodeling, toward the restoration of tissue homeostasis. This aspect is reasonable also considering the affinity of the CCs with the cartilage and of the BMSCs with the subchondral bone. On the other side, ASCs would be more useful to counteract the immune cells when placed in an inflammatory microenvironment. Finally, because these cells showed immunomodulatory ability, their potential use to facilitate the resolution of OA induced inflammation and cartilage regeneration should be advised.

Our data are in line with those reported by previous publications. In co-culture with non-polarized macrophages, CCs have already shown an enhancement in maturation (19, 40–43). Furthermore, CCs were able to shift the M1 macrophage phenotype toward the M2-like phenotype (43) and to modulate pro-inflammatory macrophage activity by reducing MHC-II expression and TNF-α secretion (44). The anti-inflammatory and pro-regenerative interaction of CCs and macrophages is particularly clinically relevant for the achievement of the joint homeostasis in a patient who undergoes cell therapy in an OA context.

In this study, we also observed the MSC ability to modulate macrophage polarization. Other scientific reports analyzed the ability of ASCs in co-culture with synovial cells to interact with macrophages, showing that the amount of synovial macrophages differently induced or down-modulated inflammatory and degradative factors (45). BMSCs previously showed immunomodulatory ability in co-culture with macrophages. These cells promoted an anti-inflammatory phenotype of macrophages, assessed in term of decrease in M1-cytokines (TNFα and IL-1β) (46, 47) and increase of M2-cytokines (CCL17, CCL22) secretion (47) or of increase in the frequency of M2-macrophages (48).

With respect to T cells, CCs already showed a reduced induction of proliferation and activation in allogeneic T cells and a potent ability to suppress allogeneic T cell proliferation, which was dependent on nitric oxide production (44).

In our study, CD4^+^ helper T cells did not proliferate, whereas CD8^+^ cytotoxic T cells showed higher survival when co-cultured with CCs and ASCs. A possible reason for this response could be the differential expression on CCs and ASCs of MHC class II and I, presenting to helper and cytotoxic T cells, respectively. In fact, these cells showed the expression of MHC class I, but not of MHC class II (49–51).

These results are interesting since lymphoid cell aggregates, containing primarily CD3^+^ T lymphocytes, were found in the synovial membrane of the 65% of patients with OA (52) and the most prevalent T cells type found in the synovium were T helper cells (CD3^+^, CD4^+^, and CD8^–^) (23, 53), acknowledged as having a pivotal role in the pathogenesis of OA (54). Cytotoxic/suppressor T cells occur sparsely and are not the predominant T cell type in the synovial aggregates of OA patients (21). Nevertheless, these cells likely shape the pathogenesis of OA, although they do not play the most important role in this disease (55). Considering these observations, the lack of promotion of CD4^+^ and the slight promotion of the CD8^+^ T cell survival mediated by CCs and ASCs reflects a non-immunogenicity of these cells. This is particularly interesting for CD4^+^ cells, which are considered predominant and active in OA infiltrates, whereas the data reported for CD8^+^ cells should be better evaluated when their role in OA pathophysiology will be better elucidated.

The main limitation of the present study resides in the fact that, as with all the co-culture *in vitro* tests, these present the intrinsic limitation of being based on pre-established models. These models are unable to faithfully represent the dynamic OA pathophysiology. Nevertheless, if compared with *in vitro* models exploiting the conditioned medium to mimic the inflammatory status, the use of co-culture tests with more cell types allows at least in part overcoming this limitation. In fact, co-culture tests add complexity and dynamicity to classical *in vitro* tests, allowing for exploration of crosstalk between cells sharing the same environment. Another limitation is the number of donors used in this study. The results need to be confirmed in a wider population, but are useful to restrict the biological fields of investigation in the OA context.

## Conclusion

In conclusion, this study is a proof-of-concept to support the idea that bioinformatics data need to be validated by means of co-culture tests, establishing in reality which pathways are activated by cell crosstalk and how much these pathways contribute in the overall outcome in the context of a biological process. In fact, although data derived by gene, miRNA and protein arrays are a precious mine of information, they can only provide an idea of the processes modulated by the cells. But, when these cells are placed in specific contexts, being competent, they respond to the stimuli they find in the environment, in turn producing mediators that stimulate both the cells themselves and those with which they interact. As a consequence, co-culture tests represent an essential step in the investigation of the effect of cell therapy in the modulation of a biological process, while the analysis of the expression of genes, miRNAs and proteins should be the tool that allows defining a modulation in a pro- or anti-inflammatory sense. Using this approach, we found that CCs, ASCs, and BMSCs are able to modulate macrophage phenotype and *T* cell survival, by promoting a general anti-inflammatory environment without inducing an inflammatory response mediated by immune cells, which usually infiltrate the synovial membrane of OA patients.

## Data availability statement

The data presented in this study are deposited in the OSF repository, accession number: https://osf.io/y96rc/?view_only=e96afba950ec469bb7cfea1162aa5672.

## Author contributions

AC: conception and design, collection and/or assembly of data, data analysis and interpretation, and manuscript writing. FL, ER, PD, and FS: collection and/or assembly of data and final approval of the manuscript. SL: collection and/or assembly of data, data analysis and interpretation, and final approval of the manuscript. LZ: provision of the study material or patients and final approval of the manuscript. MM and LG: financial support and final approval of the manuscript. All authors contributed to the article and approved the submitted version.

## Footnotes

1
http://userver.bio.uniroma1.it/apps/mienturnet/

2
www.ingenuity.com
