# Supplementary material for: Immunomodulatory potential of secretome from cartilage cells and mesenchymal stromal cells in an arthritic context: From predictive fiction toward reality
Source: Front Med (Lausanne). 2022 Oct 12;9:992386. doi: 10.3389/fmed.2022.992386 (PMC9596769; doi:10.3389/fmed.2022.992386)
Supplement: Supplementary file 1 [file Table_1.docx]

Table S1. miRNAs embedded in the EVs from CCs, ASCs and BMSCs.

|  | **CCs** | **ASCs** | **BMSCs** |
| --- | --- | --- | --- |
| **Target** | **CRT mean** | **CRT mean** | **CRT mean** |
| let-7a-3p | 24.5 |  | 24.3 |
| let-7a-5p | 14.7 | 16.0 | 13.7 |
| let-7b-5p | 13.2 | 14.2 | 12.9 |
| let-7c | 14.0 | 15.1 | 15.7 |
| let-7d-3p | 25.1 |  | 26.7 |
| let-7d-5p | 19.0 | 19.9 | 18.2 |
| let-7e-5p | 14.8 | 15.9 | 14.1 |
| let-7f-1-3p |  | 22.2 | 25.2 |
| let-7f-5p | 18.3 | 18.6 | 16.4 |
| let-7g-5p | 17.2 | 17.5 | 17.3 |
| miR-100-5p | 10.3 | 12.4 | 10.3 |
| miR-101-3p | 21.3 | 22.6 | 22.4 |
| miR-103a-3p | 17.3 | 17.4 | 16.7 |
| miR-106a-5p | 14.2 | 13.7 | 13.2 |
| miR-106b-3p | 19.7 | 19.2 | 19.1 |
| miR-106b-5p | 14.6 | 15.4 | 14.5 |
| miR-107 | 22.2 | 21.9 | 22.3 |
| miR-10a-5p | 21.0 | 17.7 | 19.9 |
| miR-10b-3p | 19.5 | 21.5 | 21.9 |
| miR-10b-5p | 20.7 | 22.5 | 22.6 |
| miR-1183 | 16.3 | 16.5 | 13.6 |
| miR-1197 | 26.3 | 25.9 |  |
| miR-1208 | 13.1 | 14.5 |  |
| miR-122-3p | 17.7 | 15.5 |  |
| miR-122-5p | 22.8 |  | 23.6 |
| miR-1226-5p | 22.1 | 21.9 | 22.4 |
| miR-1227 | 22.5 | 20.8 | 22.7 |
| miR-1233 | 17.6 | 16.4 |  |
| miR-1236-3p |  |  | 23.1 |
| miR-124-3p | 23.0 | 15.5 | 22.6 |
| miR-1244 | 25.2 | 25.2 | 26.8 |
| miR-1247-5p | 20.6 | 20.4 |  |
| miR-1253 | 20.2 |  |  |
| miR-1254 | 24.9 | 22.9 | 26.5 |
| miR-1255b-5p | 22.3 | 20.6 | 23.3 |
| miR-125a-3p | 24.7 | 24.4 | 24.7 |
| miR-125a-5p | 18.3 | 17.4 | 17.5 |
| miR-125b-1-3p |  | 21.3 | 20.9 |
| miR-125b-2-3p | 21.0 | 21.6 | 23.9 |
| miR-125b-5p | 8.6 | 9.0 | 8.9 |
| miR-1260a | 17.5 | 15.9 | 17.4 |
| miR-126-3p | 22.3 | 23.2 | 23.5 |
| miR-1265 |  | 24.1 |  |
| miR-126-5p | 24.6 | 24.6 | 25.2 |
| miR-1267 | 17.3 | 15.6 | 17.9 |
| miR-1269a | 25.6 |  |  |
| miR-1271-5p | 20.8 | 21.3 | 21.6 |
| miR-127-3p | 12.2 | 13.4 | 13.2 |
| miR-1275 | 17.2 | 16.1 | 17.5 |
| miR-127-5p | 24.2 |  |  |
| miR-1276 | 24.5 | 23.6 | 25.6 |
| miR-128 | 19.3 | 20.1 | 19.3 |
| miR-1285-3p | 21.8 | 20.7 | 24.1 |
| miR-1290 | 20.4 |  | 21.7 |
| miR-1291 | 17.2 | 18.8 | 18.8 |
| miR-129-2-3p | 22.2 | 19.2 | 20.6 |
| miR-129-5p |  | 22.5 | 22.7 |
| miR-1296 | 18.2 | 17.7 | 18.9 |
| miR-1300 (v13) | 23.7 | 23.5 | 25.1 |
| miR-1303 | 20.0 | 19.2 | 21.6 |
| miR-1304-5p | 18.8 |  | 19.7 |
| miR-130a-3p | 13.3 | 14.1 | 13.2 |
| miR-130b-3p | 14.7 | 16.5 | 15.1 |
| miR-130b-5p | 24.2 | 21.7 | 23.7 |
| miR-132-3p | 14.0 | 13.6 | 13.4 |
| miR-132-5p |  | 23.0 |  |
| miR-133a | 19.1 |  | 19.8 |
| miR-133b | 21.8 |  | 24.0 |
| miR-134 | 16.8 | 19.5 | 17.6 |
| miR-135b-3p | 17.6 | 19.5 | 18.5 |
| miR-135b-5p | 24.6 | 23.6 |  |
| miR-136-3p | 20.3 | 22.1 | 22.1 |
| miR-137 | 20.2 | 17.3 | 16.6 |
| miR-138-5p | 12.3 | 13.9 | 13.8 |
| miR-139-5p | 21.2 | 21.4 | 20.8 |
| miR-1-3p |  |  | 22.5 |
| miR-140-3p | 17.5 | 21.4 | 20.8 |
| miR-140-5p | 13.5 | 18.5 | 15.9 |
| miR-141-3p |  |  | 22.5 |
| miR-142-3p | 25.6 | 24.6 | 23.7 |
| miR-143-3p | 18.2 | 17.1 | 13.9 |
| miR-143-5p |  |  | 18.1 |
| miR-145-3p | 25.8 | 24.4 | 21.6 |
| miR-145-5p | 14.4 | 12.1 | 9.3 |
| miR-146a-5p | 19.6 | 17.5 | 18.8 |
| miR-146b-3p | 20.3 | 21.0 | 23.7 |
| miR-146b-5p | 13.7 | 15.8 | 16.7 |
| miR-148a-3p | 14.9 | 17.1 | 16.0 |
| miR-148b-3p | 19.4 | 20.5 | 19.9 |
| miR-148b-5p | 25.4 | 26.7 | 25.5 |
| miR-149-5p | 17.3 | 15.5 | 16.3 |
| miR-150-5p | 21.7 | 21.4 | 21.7 |
| miR-151a-3p | 19.0 | 19.2 | 20.0 |
| miR-151a-5p | 21.6 | 22.0 | 22.7 |
| miR-152 | 12.6 | 14.1 | 13.2 |
| miR-154-3p | 23.1 | 24.9 | 24.1 |
| miR-154-5p | 19.3 | 20.6 | 20.4 |
| miR-155-5p | 17.8 | 18.7 | 18.9 |
| miR-15a-3p | 25.0 | 23.4 | 24.5 |
| miR-15a-5p | 19.9 | 20.4 | 19.5 |
| miR-15b-3p | 21.1 |  | 20.0 |
| miR-15b-5p | 15.9 | 16.1 | 15.9 |
| miR-16-1-3p | 24.6 | 24.6 | 25.2 |
| miR-16-5p | 13.4 | 14.0 | 14.8 |
| miR-17-3p | 25.1 |  |  |
| miR-17-5p | 14.1 | 13.6 | 13.3 |
| miR-181a-3p | 24.6 | 22.9 | 23.4 |
| miR-181a-3p | 25.4 | 23.3 | 22.4 |
| miR-181a-5p | 18.9 | 16.7 | 14.7 |
| miR-181c-3p | 22.3 |  | 22.1 |
| miR-181c-5p | 21.1 | 21.9 | 20.8 |
| miR-182-3p | 23.8 |  |  |
| miR-183-3p |  | 23.4 |  |
| miR-184 | 21.8 | 22.0 | 21.8 |
| miR-185-5p | 19.5 | 20.8 | 19.4 |
| miR-186-5p | 16.4 | 17.6 | 16.5 |
| miR-187-3p | 21.9 | 17.7 | 22.2 |
| miR-18a-3p |  | 26.2 | 25.9 |
| miR-18a-5p | 20.6 | 20.7 | 19.7 |
| miR-18b-5p | 21.0 | 20.8 | 19.2 |
| miR-190a | 22.4 | 21.7 | 21.6 |
| miR-190b | 25.7 | 26.9 |  |
| miR-191-5p | 10.9 | 11.8 | 11.4 |
| miR-192-5p | 18.6 | 19.6 | 18.9 |
| miR-193a-3p | 25.4 | 24.7 | 25.1 |
| miR-193a-5p | 14.8 | 14.0 | 13.5 |
| miR-193b-3p | 10.3 | 10.4 | 10.4 |
| miR-193b-5p | 20.2 | 19.6 | 19.7 |
| miR-194-5p | 22.0 | 20.2 | 22.0 |
| miR-195-5p | 15.8 | 17.6 | 18.1 |
| miR-196b-5p | 21.3 | 23.4 | 20.7 |
| miR-197-3p | 14.6 | 13.3 | 13.6 |
| miR-198 | 22.6 | 23.0 | 24.0 |
| miR-199a-3p; miR-199b-3p | 13.7 | 13.7 | 13.5 |
| miR-199a-5p | 19.3 | 18.7 | 18.3 |
| miR-199b-5p | 20.3 | 18.2 | 19.9 |
| miR-19a-3p | 18.1 | 18.1 | 17.5 |
| miR-19b-1-5p |  | 23.9 | 23.8 |
| miR-19b-3p | 11.8 | 12.0 | 11.3 |
| miR-200a-3p | 17.5 | 15.1 | 15.2 |
| miR-200b-3p | 23.9 |  | 23.6 |
| miR-200c-3p | 21.5 |  |  |
| miR-202-3p | 24.2 | 22.7 | 20.3 |
| miR-203 | 18.4 | 21.1 | 18.8 |
| miR-204-5p | 20.0 | 17.3 | 19.1 |
| miR-205-5p |  |  | 18.8 |
| miR-206 | 25.2 | 24.5 | 25.2 |
| miR-20a-3p | 26.7 | 26.6 |  |
| miR-20a-5p |  | 12.6 | 12.2 |
| miR-20b-5p | 21.2 | 20.3 | 19.9 |
| miR-210 | 11.7 | 15.1 | 12.5 |
| miR-211-5p | 19.4 | 15.0 | 14.5 |
| miR-212-3p | 18.3 | 17.5 | 18.1 |
| miR-21-3p | 22.2 | 22.3 | 22.4 |
| miR-214-3p | 12.7 | 12.6 | 12.7 |
| miR-214-5p | 21.6 | 20.6 | 21.3 |
| miR-215 | 18.6 | 20.6 | 19.7 |
| miR-21-5p | 9.4 | 11.4 | 9.8 |
| miR-216a-5p | 23.5 |  |  |
| miR-216b-5p | 23.2 |  |  |
| miR-217-5p | 25.1 |  |  |
| miR-218-2-3p |  | 26.6 |  |
| miR-218-5p | 18.1 | 13.8 | 14.8 |
| miR-219-1-3p |  | 26.2 |  |
| miR-219-5p |  | 24.5 |  |
| miR-221-3p | 11.2 | 9.8 | 9.3 |
| miR-221-5p | 23.2 | 25.0 | 24.6 |
| miR-222-3p | 8.1 | 10.1 | 9.4 |
| miR-222-5p | 20.1 | 19.8 | 19.4 |
| miR-223-3p | 21.9 | 21.1 | 21.2 |
| miR-223-5p | 26.0 | 25.2 | 24.1 |
| miR-22-3p | 14.9 | 15.6 | 15.1 |
| miR-224-5p | 16.4 | 14.8 | 14.1 |
| miR-22-5p | 19.8 | 20.1 | 19.5 |
| miR-23a-3p | 15.3 | 16.2 | 15.6 |
| miR-23a-5p | 23.9 | 24.1 | 24.7 |
| miR-23b-3p | 14.8 | 16.3 | 15.0 |
| miR-24-2-5p | 20.3 | 20.9 | 20.8 |
| miR-24-3p | 7.2 | 8.2 | 8.1 |
| miR-25-3p | 15.0 | 15.7 | 15.3 |
| miR-25-5p | 23.1 |  |  |
| miR-26a-5p | 13.5 | 14.0 | 13.8 |
| miR-26b-5p | 15.0 | 16.3 | 15.2 |
| miR-27a-3p | 13.9 | 14.4 | 14.2 |
| miR-27a-5p | 21.4 | 22.4 | 22.2 |
| miR-27b-3p | 13.2 | 14.9 | 14.0 |
| miR-27b-5p | 21.5 | 23.6 | 22.6 |
| miR-28-3p | 15.2 | 16.2 | 14.6 |
| miR-28-5p | 15.2 | 15.7 | 14.7 |
| miR-296-3p | 23.1 | 21.7 | 22.7 |
| miR-296-5p | 16.6 | 15.3 | 14.7 |
| miR-299-5p | 21.7 | 22.6 | 22.1 |
| miR-29a-3p | 13.3 | 13.8 | 13.5 |
| miR-29a-5p | 18.1 | 18.6 | 17.7 |
| miR-29b-3p | 17.7 | 18.0 | 16.3 |
| miR-29c-3p | 14.2 | 15.2 | 15.0 |
| miR-29c-5p | 22.1 | 22.1 | 22.8 |
| miR-301a-3p | 16.8 | 18.1 | 16.9 |
| miR-301b | 21.1 | 22.7 | 21.4 |
| miR-302a-3p | 18.7 | 14.8 | 15.4 |
| miR-302c-3p | 19.6 | 23.0 | 11.2 |
| miR-302d-3p | 25.1 | 25.8 |  |
| miR-30a-3p | 16.7 | 16.8 | 15.4 |
| miR-30a-5p | 13.4 | 14.6 | 14.0 |
| miR-30b-5p | 10.7 | 11.4 | 10.7 |
| miR-30c-2-3p |  |  | 21.5 |
| miR-30c-5p | 10.5 | 11.2 | 10.5 |
| miR-30d-3p | 24.4 | 24.5 | 24.7 |
| miR-30d-5p | 16.2 | 17.2 | 17.2 |
| miR-30e-3p | 16.3 | 16.7 | 15.3 |
| miR-31-3p | 15.2 | 15.7 | 16.1 |
| miR-31-5p | 11.8 | 12.1 | 12.1 |
| miR-320a | 12.1 | 12.8 | 12.9 |
| miR-320b | 17.9 | 19.0 | 19.3 |
| miR-323-3p | 18.5 | 19.9 | 19.4 |
| miR-324-3p | 18.0 | 18.9 | 18.5 |
| miR-324-5p | 16.6 | 16.8 | 15.4 |
| miR-328 | 12.6 | 12.6 | 12.3 |
| miR-329 | 20.6 | 22.2 | 21.3 |
| miR-330-3p | 18.6 | 20.0 | 19.4 |
| miR-331-3p | 14.2 | 14.7 | 14.1 |
| miR-331-5p | 20.7 | 20.0 | 16.3 |
| miR-335-3p | 20.9 | 21.4 | 20.4 |
| miR-335-5p | 13.9 | 16.5 | 13.8 |
| miR-337-3p | 23.3 | 23.8 | 24.5 |
| miR-337-5p | 18.9 | 21.1 | 20.1 |
| miR-338-5p | 25.7 | 25.2 | 24.9 |
| miR-339-3p | 20.5 | 20.6 | 20.4 |
| miR-339-5p | 16.5 | 15.9 | 15.6 |
| miR-33a-3p | 25.7 | 24.8 | 26.6 |
| miR-340-3p | 25.8 | 26.7 | 26.7 |
| miR-340-5p | 24.0 | 25.5 | 25.1 |
| miR-342-3p | 16.4 | 15.8 | 16.2 |
| miR-345-5p | 17.9 | 18.5 | 17.8 |
| miR-346 | 18.5 | 17.6 | 19.4 |
| miR-34a-3p | 17.7 | 17.7 | 17.1 |
| miR-34a-5p | 13.1 | 12.8 | 12.2 |
| miR-34b-3p | 20.3 | 18.4 | 19.2 |
| miR-34b-5p | 23.3 | 22.1 | 22.7 |
| miR-34c-5p | 19.5 | 17.3 | 17.3 |
| miR-361-3p | 22.6 |  | 23.4 |
| miR-361-5p | 16.4 | 16.6 | 16.1 |
| miR-362-3p | 23.0 | 24.8 | 22.7 |
| miR-362-5p | 22.9 | 23.0 | 22.7 |
| miR-363-3p |  |  | 18.3 |
| miR-365a-3p; miR-365b-3p | 15.6 | 15.4 | 14.9 |
| miR-367-3p | 15.7 | 15.1 | 16.8 |
| miR-369-3p | 21.4 | 23.5 | 23.2 |
| miR-369-5p | 26.0 |  | 25.9 |
| miR-370 | 15.1 | 15.7 | 16.2 |
| miR-372 | 23.3 | 24.0 | 24.4 |
| miR-373-3p | 23.0 | 20.6 | 21.9 |
| miR-374a-5p | 17.3 | 18.2 | 17.3 |
| miR-374b-5p | 18.0 | 20.1 | 18.0 |
| miR-375 |  | 24.9 | 23.8 |
| miR-376a-3p | 13.9 | 16.1 | 15.3 |
| miR-376b | 21.7 |  | 22.0 |
| miR-376c | 13.8 | 16.2 | 16.2 |
| miR-377-3p | 22.8 |  | 24.5 |
| miR-377-5p | 24.6 | 24.1 | 25.6 |
| miR-378a-3p | 24.4 | 23.0 | 23.4 |
| miR-378a-5p | 26.0 | 24.3 | 24.2 |
| miR-379-5p | 18.1 | 19.0 | 18.8 |
| miR-380-3p | 22.9 |  | 23.2 |
| miR-380-5p | 22.8 | 24.0 | 25.7 |
| miR-381 | 20.1 | 21.4 | 22.1 |
| miR-382-5p | 12.2 | 13.5 | 13.3 |
| miR-383 |  | 20.3 |  |
| miR-409-3p | 14.0 | 14.6 | 14.7 |
| miR-409-5p | 20.7 | 21.9 | 21.7 |
| miR-410 | 16.9 | 19.2 | 18.8 |
| miR-411-3p | 25.5 | 25.2 | 25.9 |
| miR-411-5p | 16.5 | 18.5 | 17.1 |
| miR-422a | 25.3 | 23.8 | 24.4 |
| miR-423-5p | 17.3 | 17.0 | 16.5 |
| miR-424-3p | 21.9 | 19.8 | 21.1 |
| miR-424-5p | 23.5 | 19.8 | 20.7 |
| miR-425-3p | 24.2 | 23.1 | 23.7 |
| miR-425-5p | 16.2 | 17.5 | 17.4 |
| miR-431-3p | 23.0 |  |  |
| miR-431-5p | 17.7 |  | 19.5 |
| miR-432-3p | 24.6 | 22.2 |  |
| miR-432-5p | 20.2 | 21.0 | 21.4 |
| miR-433 | 19.3 | 19.6 | 20.5 |
| miR-449a | 26.1 |  |  |
| miR-449b-5p | 24.0 |  | 23.8 |
| miR-450a-5p |  | 24.5 | 24.8 |
| miR-451a | 23.4 | 22.0 | 22.0 |
| miR-452-5p | 21.3 | 21.3 | 19.6 |
| miR-454-3p |  | 22.7 | 21.6 |
| miR-455-3p | 17.8 | 19.0 | 18.3 |
| miR-455-5p | 17.6 | 20.3 | 19.2 |
| miR-483-3p |  |  | 19.3 |
| miR-483-5p | 17.2 | 16.3 | 14.5 |
| miR-484 | 12.4 | 12.8 | 12.2 |
| miR-485-3p | 19.9 | 20.6 | 20.1 |
| miR-485-5p | 18.8 |  | 19.4 |
| miR-486-5p | 22.1 |  | 21.1 |
| miR-487a | 22.4 | 22.9 | 23.5 |
| miR-487b | 19.7 | 19.8 | 21.3 |
| miR-488-5p | 24.2 | 23.2 |  |
| miR-489 | 22.3 |  | 20.8 |
| miR-491-5p | 19.1 | 22.5 | 19.6 |
| miR-493-3p | 20.8 | 22.8 | 21.9 |
| miR-494 | 17.0 | 18.7 | 18.7 |
| miR-495 | 16.3 | 16.7 | 17.9 |
| miR-496 | 25.8 |  |  |
| miR-497-5p | 20.2 | 21.7 | 23.0 |
| miR-500a-3p | 23.3 | 23.3 | 24.4 |
| miR-500a-5p | 21.7 | 21.1 | 21.5 |
| miR-501-3p | 20.9 | 21.1 | 20.9 |
| miR-501-5p |  | 21.6 | 20.0 |
| miR-502-3p | 21.2 | 21.7 | 21.8 |
| miR-502-5p | 21.7 | 21.5 | 21.7 |
| miR-503 | 23.1 | 20.2 | 21.6 |
| miR-505-3p | 20.4 | 20.2 | 20.5 |
| miR-505-5p | 24.1 | 22.8 | 24.6 |
| miR-509-5p | 21.5 | 21.3 | 16.5 |
| miR-512-3p | 19.3 | 21.6 |  |
| miR-516a-3p; miR-516b-3p | 25.8 | 24.2 | 24.3 |
| miR-517c-3p | 26.8 | 20.8 |  |
| miR-518d-3p | 22.3 | 23.0 |  |
| miR-520b | 23.0 |  | 25.1 |
| miR-520c-3p | 24.7 | 25.5 | 25.4 |
| miR-520d-3p |  | 26.8 | 21.6 |
| miR-520e | 20.9 | 19.3 |  |
| miR-520f | 25.1 | 21.0 | 22.5 |
| miR-523-3p | 15.9 |  | 13.6 |
| miR-524-3p | 19.1 |  |  |
| miR-526b-5p | 23.6 | 20.7 |  |
| miR-532-3p | 17.6 | 20.1 | 17.4 |
| miR-532-5p | 15.7 | 17.7 | 15.7 |
| miR-539-5p | 18.4 | 21.4 | 20.4 |
| miR-541-5p |  | 22.1 |  |
| miR-542-3p | 23.1 | 23.1 | 24.4 |
| miR-542-5p | 24.9 | 23.9 | 24.3 |
| miR-543 | 19.2 | 21.0 | 20.5 |
| miR-545-3p | 23.1 | 23.6 | 25.6 |
| miR-548a-3p |  | 23.9 | 26.5 |
| miR-548am-5p; | 22.1 | 16.9 | 22.3 |
| miR-548b-3p |  | 20.1 | 22.3 |
| miR-548d-5p | 22.2 | 23.3 | 22.8 |
| miR-551b-3p |  | 15.0 | 24.4 |
| miR-551b-5p |  |  | 25.0 |
| miR-564 | 20.8 | 19.5 | 22.9 |
| miR-566 |  | 16.4 | 15.8 |
| miR-572 | 21.7 | 21.2 | 21.8 |
| miR-574-3p | 11.7 | 15.6 | 12.0 |
| miR-576-3p | 22.3 | 22.9 | 21.7 |
| miR-576-5p | 21.7 | 21.7 | 21.9 |
| miR-579 | 22.7 | 22.6 | 22.5 |
| miR-580 |  |  | 17.0 |
| miR-589-3p | 23.7 | 22.7 | 22.7 |
| miR-590-3p | 26.4 | 26.0 | 24.8 |
| miR-590-5p | 18.9 | 19.6 | 18.5 |
| miR-591 |  | 24.2 |  |
| miR-597 | 22.5 | 21.9 | 21.6 |
| miR-598 | 24.3 |  | 26.0 |
| miR-601 | 22.9 | 23.5 |  |
| miR-604 | 26.6 |  |  |
| miR-605 | 23.4 | 20.7 | 23.4 |
| miR-615-3p | 17.3 | 14.9 | 18.0 |
| miR-615-5p | 21.6 | 23.1 | 21.7 |
| miR-616-3p | 23.0 | 23.1 | 23.4 |
| miR-616-5p | 23.7 | 23.6 | 24.9 |
| miR-617 |  |  | 26.3 |
| miR-622 | 25.2 | 24.1 |  |
| miR-623 | 20.7 | 19.6 | 14.7 |
| miR-624-5p | 25.1 | 22.9 | 25.2 |
| miR-625-3p | 22.7 | 20.9 | 22.5 |
| miR-625-5p | 25.3 | 25.3 | 25.8 |
| miR-628-3p | 25.4 | 25.1 | 25.6 |
| miR-628-5p | 23.7 | 22.8 | 24.0 |
| miR-629-3p | 23.2 | 22.6 | 22.3 |
| miR-629-5p | 25.3 | 24.6 | 24.7 |
| miR-635 |  | 24.6 |  |
| miR-636 | 15.2 | 20.1 | 10.1 |
| miR-638 | 19.9 | 19.5 | 26.8 |
| miR-641 | 26.1 |  | 20.0 |
| miR-642a-5p | 18.8 | 21.3 | 20.5 |
| miR-645 | 21.6 | 22.9 | 22.6 |
| miR-648 | 20.7 | 19.3 | 10.4 |
| miR-650 | 21.3 | 19.8 |  |
| miR-652-3p | 20.2 | 22.7 | 21.3 |
| miR-654-3p | 22.7 | 21.3 | 23.5 |
| miR-654-5p | 16.5 | 19.7 | 16.6 |
| miR-655 | 21.8 | 21.6 | 22.7 |
| miR-656 | 22.8 | 21.9 | 24.1 |
| miR-659-3p | 20.8 | 19.2 | 17.4 |
| miR-660-5p | 16.4 | 17.8 | 16.7 |
| miR-661 | 17.9 | 17.0 | 19.0 |
| miR-662 |  | 18.3 | 15.0 |
| miR-663b | 15.9 | 15.6 | 15.4 |
| miR-664-3p | 19.5 | 19.0 | 20.1 |
| miR-671-3p | 22.0 | 19.9 | 21.9 |
| miR-708-5p | 22.7 | 20.5 | 16.3 |
| miR-7-1-3p | 18.0 | 18.4 | 17.8 |
| miR-720 | 13.3 | 12.0 | 11.8 |
| miR-744-3p | 24.1 | 23.2 | 25.1 |
| miR-744-5p | 17.9 | 19.6 | 17.2 |
| miR-758 | 22.2 | 18.5 | 22.6 |
| miR-7-5p | 24.4 | 24.5 | 22.6 |
| miR-765 |  | 22.0 |  |
| miR-766-3p | 18.8 | 17.8 | 19.9 |
| miR-769-5p |  | 20.9 | 21.2 |
| miR-770-5p |  | 14.2 |  |
| miR-874 | 18.9 |  | 19.7 |
| miR-875-5p | 26.1 |  | 26.5 |
| miR-885-5p | 22.3 | 20.0 | 22.6 |
| miR-886-3p (v15) | 15.8 | 18.7 | 16.3 |
| miR-886-5p (v15) | 16.5 | 19.1 | 16.9 |
| miR-887 | 21.0 |  |  |
| miR-888-5p | 23.7 | 21.8 |  |
| miR-889 | 21.1 | 22.5 | 22.9 |
| miR-92a-3p | 11.5 | 22.3 | 11.1 |
| miR-93-3p | 21.3 | 21.8 | 21.6 |
| miR-93-5p | 16.8 | 16.5 | 16.2 |
| miR-939 | 17.9 | 15.9 | 17.9 |
| miR-942 | 21.2 | 21.0 | 20.7 |
| miR-943 | 23.4 | 22.1 |  |
| miR-95 | 23.9 | 14.3 | 26.1 |
| miR-9-5p | 22.2 | 18.0 | 23.2 |
| miR-98 | 17.9 | 20.0 | 17.7 |
| miR-99a-3p | 21.6 | 23.0 | 24.7 |
| miR-99a-5p | 10.3 | 17.2 | 10.3 |
| miR-99b-3p | 22.4 | 21.7 | 22.8 |
| miR-99b-5p | 12.9 | 15.3 | 11.9 |
| hsmiR-1180 | 22.0 | 21.4 | 21.9 |
