# Supplementary material for: Immunomodulatory potential of secretome from cartilage cells and mesenchymal stromal cells in an arthritic context: From predictive fiction toward reality
Source: Front Med (Lausanne). 2022 Oct 12;9:992386. doi: 10.3389/fmed.2022.992386 (PMC9596769; doi:10.3389/fmed.2022.992386)
Supplement: Supplementary file 2 [file Table_2.docx]

Table S2: Target genes of miRNAs embedded only in the EVs from CCs.

| **Gene Symbol** | **p-value** | **Number of interactions** | **microRNAs** |
| --- | --- | --- | --- |
| ZNF33A | 0.000038 | 4 | miR-25-5p, miR-604, miR-1253, miR-17-3p |
| HOXA13 | 0.001671 | 4 | miR-217, miR-496, miR-17-3p, miR-449a |
| SHOC2 | 0.052011 | 3 | miR-200c-3p, miR-127-5p, miR-449a |
| MDM4 | 0.038240 | 3 | miR-449a, miR-25-5p, miR-17-3p |
| RNF11 | 0.036367 | 3 | miR-1253, miR-17-3p, miR-496 |
| TMEM189 | 0.032774105 | 3 | miR-1253, miR-496, miR-604 |
| TMEM189-UBE2V1 | 0.032194957 | 3 | miR-1253, miR-496, miR-604 |
| UBE2V1 | 0.031621461 | 3 | miR-1253, miR-496, miR-604 |
| SETD1B | 0.029934889 | 3 | miR-127-5p, miR-17-3p, miR-1253 |
| E2F3 | 0.026197354 | 3 | miR-449a, miR-217, miR-200c-3p |
| CRKL | 0.023696963 | 3 | miR-17-3p, miR-1253, miR-200c-3p |
| PTEN | 0.021797868 | 3 | miR-217, miR-17-3p, miR-200c-3p |
| PTBP1 | 0.012263811 | 3 | miR-17-3p, miR-604, miR-127-5p |
| ARHGAP1 | 0.006205471 | 3 | miR-25-5p, miR-1253, miR-449a |
| SLC4A2 | 0.001673586 | 3 | miR-449a, miR-1253, miR-217 |
| SIRT1 | 0.000689001 | 3 | miR-217, miR-449a, miR-200c-3p |
| PRKCZ | 2.31036E-05 | 2 | miR-200c-3p, miR-25-5p |
| ZNF85 | 0.054716009 | 2 | miR-1253, miR-604 |
| KRAS | 0.053444783 | 2 | miR-217, miR-200c-3p |
| RALY | 0.053444783 | 2 | miR-127-5p, miR-449a |
| RAP2C | 0.053444783 | 2 | miR-200c-3p, miR-496 |
| TCF7L2 | 0.053444783 | 2 | miR-217, miR-200c-3p |
| RAB11B | 0.052184789 | 2 | miR-25-5p, miR-217 |
| STX16 | 0.050936154 | 2 | miR-17-3p, miR-200c-3p |
| ANKRD33B | 0.048473462 | 2 | miR-200c-3p, miR-1253 |
| BPNT1 | 0.044867779 | 2 | miR-17-3p, miR-25-5p |
| MTDH | 0.044867779 | 2 | miR-200c-3p, miR-217 |
| PRR13 | 0.044867779 | 2 | miR-25-5p, miR-127-5p |
| ABCB7 | 0.043689961 | 2 | miR-17-3p, miR-25-5p |
| BCL2 | 0.043689961 | 2 | miR-449a, miR-200c-3p |
| FOXO1 | 0.043689961 | 2 | miR-1253, miR-200c-3p |
| TFRC | 0.042524399 | 2 | miR-182-3p, miR-17-3p |
| HIST1H2AG | 0.040230566 | 2 | miR-1269a, miR-25-5p |
| TMEM246 | 0.037987341 | 2 | miR-217, miR-449a |
| MAST3 | 0.036885042 | 2 | miR-449a, miR-25-5p |
| VGLL4 | 0.0357958 | 2 | miR-1253, miR-17-3p |
| ZNF644 | 0.03471975 | 2 | miR-604, miR-449a |
| RRAGD | 0.03365703 | 2 | miR-449a, miR-1253 |
| SEC61A1 | 0.032607779 | 2 | miR-1253, miR-449a |
| MOCS3 | 0.031572134 | 2 | miR-182-3p, miR-25-5p |
| PLCG1 | 0.030550237 | 2 | miR-200c-3p, miR-217 |
| PSD3 | 0.030550237 | 2 | miR-182-3p, miR-200c-3p |
| MFSD8 | 0.02756844 | 2 | miR-200c-3p, miR-449a |
| CAB39 | 0.025651916 | 2 | miR-200c-3p, miR-1269a |
| MTRNR2L1 | 0.024715489 | 2 | miR-127-5p, miR-431-3p |
| PABPC1 | 0.023793813 | 2 | miR-17-3p, miR-200c-3p |
| NOTCH1 | 0.019411876 | 2 | miR-449a, miR-200c-3p |
| SRSF11 | 0.019411876 | 2 | miR-127-5p, miR-217 |
| CCNE2 | 0.018581825 | 2 | miR-449a, miR-200c-3p |
| ITGB3 | 0.018581825 | 2 | miR-449a, miR-17-3p |
| MDC1 | 0.01393871 | 2 | miR-17-3p, miR-1253 |
| KNL1 | 0.01322251 | 2 | miR-127-5p, miR-25-5p |
| LHFPL3 | 0.01322251 | 2 | miR-1269a, miR-524-3p |
| RPS6KB1 | 0.01322251 | 2 | miR-1253, miR-200c-3p |
| TRIM56 | 0.01322251 | 2 | miR-1253, miR-200c-3p |
| DENND2C | 0.012523202 | 2 | miR-127-5p, miR-25-5p |
| TMEM63A | 0.012523202 | 2 | miR-1253, miR-217 |
| ATP6V1E1 | 0.011175908 | 2 | miR-200c-3p, miR-449a |
| GSTO2 | 0.011175908 | 2 | miR-182-3p, miR-17-3p |
| CKAP4 | 0.0092857 | 2 | miR-217, miR-25-5p |
| DUSP1 | 0.0092857 | 2 | miR-200c-3p, miR-182-3p |
| MRVI1 | 0.008691147 | 2 | miR-604, miR-1253 |
| DNAJC3 | 0.005992251 | 2 | miR-127-5p, miR-200c-3p |
| MALL | 0.005508428 | 2 | miR-17-3p, miR-25-5p |
| ZDHHC16 | 0.005508428 | 2 | miR-449a, miR-1253 |
| PPL | 0.005043657 | 2 | miR-127-5p, miR-217 |
| PLA2G2D | 0.00417196 | 2 | miR-449a, miR-1253 |
| SNX10 | 0.00417196 | 2 | miR-127-5p, miR-496 |
| SYT9 | 0.003765384 | 2 | miR-127-5p, miR-1253 |
| KDR | 0.003378557 | 2 | miR-17-3p, miR-200c-3p |
