# Supplementary material for: Immunomodulatory potential of secretome from cartilage cells and mesenchymal stromal cells in an arthritic context: From predictive fiction toward reality
Source: Front Med (Lausanne). 2022 Oct 12;9:992386. doi: 10.3389/fmed.2022.992386 (PMC9596769; doi:10.3389/fmed.2022.992386)
Supplement: Supplementary file 3 [file Table_3.docx]

Table S3: Target genes of miRNAs embedded only in the EVs from ASCs.

| **Gene Symbol** | **p-value** | **Number of interactions** | **microRNAs** |
| --- | --- | --- | --- |
| CELF1 | 0.029566 | 3 | miR-541-5p. miR-635. miR-765 |
| CDK6 | 0.022596 | 3 | miR-183-3p. miR-541-5p. miR-765 |
| ZNF117 | 0.004506 | 3 | miR-770-5p. miR-183-3p. miR-218-2-3p |
| ZNF208 | 0.000404 | 3 | miR-770-5p. miR-183-3p. miR-218-2-3p |
| YWHAG | 0.000162 | 3 | miR-132-5p. miR-770-5p. miR-765 |
| IFNAR2 | 0.051746 | 2 | miR-635. miR-1265 |
| NEGR1 | 0.045861 | 2 | miR-183-3p. miR-765 |
| ZNF99 | 0.041042 | 2 | miR-183-3p. miR-770-5p |
| MEX3A | 0.040260 | 2 | miR-765. miR-541-5p |
| MLF2 | 0.037949 | 2 | miR-1265. miR-765 |
| FAM217B | 0.036440 | 2 | miR-770-5p. miR-1265 |
| GFPT1 | 0.033496 | 2 | miR-183-3p. miR-541-5p |
| ZFP69B | 0.033496 | 2 | miR-770-5p. miR-218-2-3p |
| ZNF682 | 0.029962 | 2 | miR-183-3p. miR-770-5p |
| NDRG1 | 0.029276 | 2 | miR-1265. miR-765 |
| NRF1 | 0.027922 | 2 | miR-765. miR-183-3p |
| ZNF138 | 0.022781 | 2 | miR-183-3p. miR-770-5p |
| ACVR1B | 0.022170 | 2 | miR-541-5p. miR-765 |
| MIGA2 | 0.020970 | 2 | miR-1265. miR-765 |
| CUL3 | 0.019224 | 2 | miR-591. miR-1265 |
| NLGN2 | 0.019224 | 2 | miR-765. miR-132-5p |
| GTF2F1 | 0.018097 | 2 | miR-1265, miR-765 |
| CNNM2 | 0.017545 | 2 | miR-635, miR-765 |
| ZNF83 | 0.011980 | 2 | miR-770-5p, miR-218-2-3p |
| BASP1 | 0.011522 | 2 | miR-1265, miR-635 |
| MTRNR2L10 | 0.011071 | 2 | miR-635, miR-1265 |
| CS | 0.009769 | 2 | miR-765, miR-770-5p |
| ASTN2 | 0.008943 | 2 | miR-591, miR-765 |
| MTRNR2L3 | 0.008943 | 2 | miR-635, miR-1265 |
| SMYD1 | 0.007767 | 2 | miR-183-3p, miR-765 |
| ZNF675 | 0.007025 | 2 | miR-183-3p, miR-770-5p |
| MTRNR2L8 | 0.005647 | 2 | miR-541-5p, miR-183-3p |
| TMEM248 | 0.004706 | 2 | miR-218-2-3p, miR-541-5p |
| KIF5A | 0.003069 | 2 | miR-765, miR-1265 |
| ZFAND3 | 0.002376 | 2 | miR-770-5p, miR-541-5p |
| ZNF254 | 0.001585 | 2 | miR-183-3p, miR-770-5p |
