# Supplementary material for: Immunomodulatory potential of secretome from cartilage cells and mesenchymal stromal cells in an arthritic context: From predictive fiction toward reality
Source: Front Med (Lausanne). 2022 Oct 12;9:992386. doi: 10.3389/fmed.2022.992386 (PMC9596769; doi:10.3389/fmed.2022.992386)
Supplement: Supplementary file 4 [file Table_4.docx]

Table S4: Target genes of miRNAs embedded only in the EVs from BMSCs.

| **Gene Symbol** | **p-value** | **Number of interactions** | **microRNAs** |
| --- | --- | --- | --- |
| TMEM239 | 0.000096 | 4 | miR-363-3p, miR-30c-2-3p, miR-205-5p, miR-483-3p |
| PGAM4 | 0.000077 | 4 | miR-143-5p, miR-141-3p, miR-363-3p, miR-483-3p |
| PRRG4 | 0.000741 | 4 | miR-30c-2-3p, miR-363-3p, miR-205-5p, miR-483-3p |
| ANGPTL7 | 0.000029 | 3 | miR-205-5p, miR-483-3p, miR-141-3p |
| SOD2 | 0.036747 | 3 | miR-483-3p, miR-551b-5p, miR-30c-2-3p |
| ZNF264 | 0.029018 | 3 | miR-363-3p, miR-143-5p, miR-30c-2-3p |
| NUDT3 | 0.015440 | 3 | miR-617, miR-30c-2-3p, miR-143-5p |
| SNRPD1 | 0.007300 | 3 | miR-363-3p, miR-551b-5p, miR-30c-2-3p |
| RBMS2 | 0.005942 | 3 | miR-143-5p, miR-30c-2-3p, miR-363-3p |
| TFDP2 | 0.004642 | 3 | miR-141-3p, miR-30c-2-3p, miR-143-5p |
| SMU1 | 0.003010 | 3 | miR-30c-2-3p, miR-363-3p, miR-551b-5p |
| RAP2B | 0.002612 | 3 | miR-205-5p, miR-30c-2-3p, miR-143-5p |
| SMAD4 | 0.001919 | 3 | miR-483-3p, miR-205-5p, miR-551b-5p |
| GID4 | 0.001459 | 3 | miR-363-3p, miR-143-5p, miR-617 |
| YME1L1 | 0.001165 | 3 | miR-143-5p, miR-141-3p, miR-30c-2-3p |
| OSBPL10 | 0.001120 | 3 | miR-143-5p, miR-30c-2-3p, miR-483-3p |
| PHLPP2 | 0.000873 | 3 | miR-141-3p, miR-205-5p, miR-363-3p |
| YWHAB | 0.000836 | 3 | miR-551b-5p, miR-143-5p, miR-30c-2-3p |
| GM2A | 0.000291 | 3 | miR-363-3p, miR-205-5p, miR-483-3p |
| SESN3 | 0.053892 | 2 | miR-363-3p, miR-143-5p |
| LPP | 0.053119 | 2 | miR-617, miR-141-3p |
| PTEN | 0.053119 | 2 | miR-141-3p, miR-205-5p |
| BRI3BP | 0.051587 | 2 | miR-551b-5p, miR-30c-2-3p |
| PARD6B | 0.051587 | 2 | miR-205-5p, miR-363-3p |
| PHF12 | 0.051587 | 2 | miR-205-5p, miR-483-3p |
| BCL2L11 | 0.050827 | 2 | miR-363-3p, miR-30c-2-3p |
| FAM83F | 0.050827 | 2 | miR-483-3p, miR-143-5p |
| MAZ | 0.050827 | 2 | miR-30c-2-3p, miR-483-3p |
| SMC1A | 0.050827 | 2 | miR-483-3p, miR-30c-2-3p |
| HS3ST1 | 0.050071 | 2 | miR-483-3p, miR-143-5p |
| TIAL1 | 0.050071 | 2 | miR-143-5p, miR-30c-2-3p |
| TRUB2 | 0.049321 | 2 | miR-143-5p, miR-30c-2-3p |
| HNRNPU | 0.048574 | 2 | miR-551b-5p, miR-30c-2-3p |
| LAX1 | 0.048574 | 2 | miR-30c-2-3p, miR-363-3p |
| NPR1 | 0.047832 | 2 | miR-483-3p, miR-30c-2-3p |
| PLEKHA1 | 0.047095 | 2 | miR-363-3p, miR-30c-2-3p |
| UGGT1 | 0.046362 | 2 | miR-143-5p, miR-30c-2-3p |
| WASL | 0.046362 | 2 | miR-363-3p, miR-143-5p |
| ZNF805 | 0.046362 | 2 | miR-141-3p, miR-143-5p |
| MYLIP | 0.044910 | 2 | miR-363-3p, miR-143-5p |
| GINM1 | 0.044190 | 2 | miR-143-5p, miR-30c-2-3p |
| LRIG2 | 0.044190 | 2 | miR-30c-2-3p, miR-143-5p |
| TMEM120B | 0.044190 | 2 | miR-30c-2-3p, miR-143-5p |
| HOXA13 | 0.042766 | 2 | miR-551b-5p, miR-363-3p |
| MRO | 0.042766 | 2 | miR-363-3p, miR-30c-2-3p |
| NKAP | 0.041360 | 2 | miR-30c-2-3p, miR-363-3p |
| SHISA9 | 0.041360 | 2 | miR-551b-5p, miR-483-3p |
| TNFRSF10B | 0.041360 | 2 | miR-30c-2-3p, miR-141-3p |
| CREB1 | 0.039974 | 2 | miR-205-5p, miR-363-3p |
| SNRPD3 | 0.039974 | 2 | miR-363-3p, miR-30c-2-3p |
| FUT11 | 0.039288 | 2 | miR-363-3p, miR-141-3p |
| ATF7IP | 0.038606 | 2 | miR-363-3p, miR-30c-2-3p |
| MRPS16 | 0.037930 | 2 | miR-30c-2-3p, miR-363-3p |
| MTMR10 | 0.036592 | 2 | miR-30c-2-3p, miR-363-3p |
| LRRC3C | 0.035273 | 2 | miR-143-5p, miR-30c-2-3p |
| DDI2 | 0.034622 | 2 | miR-363-3p, miR-551b-5p |
| ZNF317 | 0.034622 | 2 | miR-30c-2-3p, miR-363-3p |
| IGF1 | 0.033975 | 2 | miR-483-3p, miR-30c-2-3p |
| AR | 0.033333 | 2 | miR-205-5p, miR-30c-2-3p |
| GPR137C | 0.033333 | 2 | miR-30c-2-3p, miR-483-3p |
| MARCH6 | 0.032697 | 2 | miR-141-3p, miR-30c-2-3p |
| NPLOC4 | 0.032697 | 2 | miR-143-5p, miR-30c-2-3p |
| PPIC | 0.032066 | 2 | miR-363-3p, miR-143-5p |
| TRAF3IP2 | 0.031439 | 2 | miR-143-5p, miR-30c-2-3p |
| REEP3 | 0.030818 | 2 | miR-143-5p, miR-30c-2-3p |
| ALG14 | 0.030202 | 2 | miR-363-3p, miR-143-5p |
| RBM43 | 0.029592 | 2 | miR-143-5p, miR-30c-2-3p |
| SPRYD4 | 0.028986 | 2 | miR-141-3p, miR-363-3p |
| MAPK14 | 0.027791 | 2 | miR-141-3p, miR-205-5p |
| TMEM33 | 0.027202 | 2 | miR-30c-2-3p, miR-363-3p |
| ECE1 | 0.026618 | 2 | miR-551b-5p, miR-143-5p |
| YES1 | 0.026618 | 2 | miR-205-5p, miR-30c-2-3p |
| AGAP9 | 0.026039 | 2 | miR-30c-2-3p, miR-143-5p |
| ABCF1 | 0.025466 | 2 | miR-143-5p, miR-205-5p |
| RBBP4 | 0.025466 | 2 | miR-205-5p, miR-30c-2-3p |
| ZNF281 | 0.025466 | 2 | miR-551b-5p, miR-143-5p |
| ZNF772 | 0.025466 | 2 | miR-143-5p, miR-363-3p |
| AAED1 | 0.024898 | 2 | miR-30c-2-3p, miR-363-3p |
| EVI5 | 0.024898 | 2 | miR-551b-5p, miR-30c-2-3p |
| ZNF607 | 0.024898 | 2 | miR-30c-2-3p, miR-363-3p |
| MCF2L2 | 0.024336 | 2 | miR-30c-2-3p, miR-363-3p |
| BCL9L | 0.023780 | 2 | miR-205-5p, miR-30c-2-3p |
| FBXW2 | 0.023780 | 2 | miR-30c-2-3p, miR-363-3p |
| MRPL17 | 0.023780 | 2 | miR-143-5p, miR-30c-2-3p |
| KLF7 | 0.022683 | 2 | miR-30c-2-3p, miR-617 |
| RAN | 0.022683 | 2 | miR-483-3p, miR-205-5p |
| UBE2Z | 0.022683 | 2 | miR-363-3p, miR-205-5p |
| CD180 | 0.021609 | 2 | miR-363-3p, miR-30c-2-3p |
| PRICKLE1 | 0.021609 | 2 | miR-30c-2-3p, miR-551b-5p |
| ZNF621 | 0.021609 | 2 | miR-143-5p, miR-141-3p |
| BAMBI | 0.021080 | 2 | miR-143-5p, miR-205-5p |
| EIF5A2 | 0.020557 | 2 | miR-551b-5p, miR-363-3p |
| PGAM5 | 0.020557 | 2 | miR-30c-2-3p, miR-143-5p |
| TJAP1 | 0.020557 | 2 | miR-30c-2-3p, miR-483-3p |
| CAPZB | 0.020040 | 2 | miR-30c-2-3p, miR-363-3p |
| PLEKHB2 | 0.020040 | 2 | miR-30c-2-3p, miR-143-5p |
| ZDHHC24 | 0.020040 | 2 | miR-30c-2-3p, miR-363-3p |
| FAM229B | 0.019529 | 2 | miR-143-5p, miR-483-3p |
| YTHDF1 | 0.019529 | 2 | miR-617, miR-551b-5p |
| CHORDC1 | 0.019023 | 2 | miR-30c-2-3p, miR-551b-5p |
| ZNF695 | 0.019023 | 2 | miR-363-3p, miR-30c-2-3p |
| BAK1 | 0.017542 | 2 | miR-30c-2-3p, miR-363-3p |
| PAQR5 | 0.017060 | 2 | miR-143-5p, miR-30c-2-3p |
| APOBEC3F | 0.016583 | 2 | miR-30c-2-3p, miR-363-3p |
| AQR | 0.016583 | 2 | miR-30c-2-3p, miR-551b-5p |
| BUB1 | 0.016583 | 2 | miR-30c-2-3p, miR-551b-5p |
| ZNF770 | 0.016583 | 2 | miR-551b-5p, miR-30c-2-3p |
| PKNOX1 | 0.016113 | 2 | miR-143-5p, miR-363-3p |
| TPI1 | 0.016113 | 2 | miR-30c-2-3p, miR-483-3p |
| GOLGA3 | 0.015649 | 2 | miR-363-3p, miR-30c-2-3p |
| ZSCAN29 | 0.015649 | 2 | miR-30c-2-3p, miR-143-5p |
| PSMD11 | 0.014739 | 2 | miR-30c-2-3p, miR-141-3p |
| SEC63 | 0.014739 | 2 | miR-30c-2-3p, miR-483-3p |
| TRAF3IP1 | 0.014739 | 2 | miR-205-5p, miR-143-5p |
| DPYSL5 | 0.014294 | 2 | miR-30c-2-3p, miR-483-3p |
| MSL2 | 0.014294 | 2 | miR-483-3p, miR-30c-2-3p |
| SLC25A32 | 0.014294 | 2 | miR-363-3p, miR-483-3p |
| SRD5A1 | 0.013421 | 2 | miR-30c-2-3p, miR-205-5p |
| ZNF277 | 0.013421 | 2 | miR-30c-2-3p, miR-363-3p |
| EXTL3 | 0.012993 | 2 | miR-617, miR-30c-2-3p |
| MED7 | 0.012993 | 2 | miR-363-3p, miR-143-5p |
| VMA21 | 0.012993 | 2 | miR-363-3p, miR-143-5p |
| FOPNL | 0.012572 | 2 | miR-363-3p, miR-30c-2-3p |
| PLXDC2 | 0.012572 | 2 | miR-143-5p, miR-617 |
| UQCRFS1 | 0.012158 | 2 | miR-141-3p, miR-363-3p |
| SCD5 | 0.011749 | 2 | miR-30c-2-3p, miR-141-3p |
| XPOT | 0.011749 | 2 | miR-141-3p, miR-205-5p |
| AFF1 | 0.011347 | 2 | miR-205-5p, miR-143-5p |
| LETM1 | 0.011347 | 2 | miR-30c-2-3p, miR-363-3p |
| MED13 | 0.011347 | 2 | miR-141-3p, miR-205-5p |
| B4GALT5 | 0.010951 | 2 | miR-205-5p, miR-551b-5p |
| CPEB3 | 0.010562 | 2 | miR-205-5p, miR-363-3p |
| NUP43 | 0.010562 | 2 | miR-30c-2-3p, miR-363-3p |
| PPARA | 0.010562 | 2 | miR-141-3p, miR-30c-2-3p |
| ZCCHC2 | 0.010562 | 2 | miR-551b-5p, miR-617 |
| GCNT3 | 0.010179 | 2 | miR-363-3p, miR-551b-5p |
| LYN | 0.010179 | 2 | miR-205-5p, miR-143-5p |
| PIK3C2B | 0.009803 | 2 | miR-551b-5p, miR-483-3p |
| TRAPPC2B | 0.009803 | 2 | miR-141-3p, miR-30c-2-3p |
| LRAT | 0.008363 | 2 | miR-551b-5p, miR-143-5p |
| STXBP2 | 0.008363 | 2 | miR-141-3p, miR-143-5p |
| SLC7A2 | 0.007683 | 2 | miR-30c-2-3p, miR-205-5p |
| EXOC5 | 0.007352 | 2 | miR-363-3p, miR-551b-5p |
| TRAM2 | 0.007029 | 2 | miR-30c-2-3p, miR-363-3p |
| ZEB1 | 0.007029 | 2 | miR-141-3p, miR-205-5p |
| ERBB3 | 0.006100 | 2 | miR-205-5p, miR-143-5p |
| TSPAN31 | 0.006100 | 2 | miR-363-3p, miR-143-5p |
| ZNF321P | 0.005515 | 2 | miR-143-5p, miR-617 |
| EID2B | 0.004957 | 2 | miR-205-5p, miR-363-3p |
| DENND2C | 0.004689 | 2 | miR-143-5p, miR-363-3p |
| ACAA2 | 0.004428 | 2 | miR-30c-2-3p, miR-143-5p |
| KCNJ10 | 0.004428 | 2 | miR-205-5p, miR-617 |
| DNPEP | 0.003927 | 2 | miR-143-5p, miR-483-3p |
| FMN1 | 0.003927 | 2 | miR-363-3p, miR-143-5p |
| CTGF | 0.003455 | 2 | miR-205-5p, miR-483-3p |
| ZEB2 | 0.003455 | 2 | miR-141-3p, miR-205-5p |
| S1PR1 | 0.003229 | 2 | miR-363-3p, miR-483-3p |
| GOT1 | 0.002800 | 2 | miR-205-5p, miR-483-3p |
| AGMO | 0.002031 | 2 | miR-143-5p, miR-483-3p |
| SLC39A14 | 0.001857 | 2 | miR-205-5p, miR-363-3p |
| VAC14 | 0.001532 | 2 | miR-141-3p, miR-30c-2-3p |
| KCTD16 | 0.001102 | 2 | miR-205-5p, miR-483-3p |
| MED19 | 0.000853 | 2 | miR-363-3p, miR-551b-5p |
| PRR15 | 0.000368 | 2 | miR-205-5p, miR-483-3p |
